# Enhanced lymphocyte infiltration in the liver of LDL receptor and Myeloid Differentiation 1 double knockout mice on high fat diet

**DOI:** 10.1038/s41598-025-21070-x

**Published:** 2025-10-23

**Authors:** Mrityunjoy Biswas, Susumu Tomono, Kenji Kasai, Hidekazu Takagi, Masanori Inui, Bristy Basak, Fumiaki Nagaoka, Tatsuya Yamazaki, Naoko Morita, Akinori Okumura, Sachiko Akashi-Takamura

**Affiliations:** 1 Department of Microbiology and Immunology, Aichi Medical University, School of Medicine, 1-1 Yazakokarimata, Nagakute, Aichi 480-1195 Japan; 2 Department of Pathology, Aichi Medical University, School of Medicine, Nagakute, Aichi Japan

**Keywords:** Myeloid differentiation 1, *ly86*, MD-1, RP105, Atherosclerosis, B-cell infiltration, Immunology, Molecular biology, Molecular medicine

## Abstract

Myeloid differentiation 1 (MD-1; gene name: *ly86*), a glycoprotein that forms a complex with radioprotective protein 105 (RP105), is involved in the regulation of inflammation, obesity, and insulin resistance. Previous reports have shown an exacerbation of cardiac pathology in MD-1 deficient mice, including pressure overload-induced cardiac remodeling and high fat diet (HFD) -induced inflammatory atrial fibrosis. Furthermore, MD-1 expression is upregulated in atherosclerosis, as indicated by DEG database analysis, and is present in human atherosclerotic plaques. However, its involvement in atherosclerosis is unclear. In this study, we analyzed the effect of MD-1 deficiency on the development of HFD-induced atherosclerosis in littermates of low-density lipoprotein (LDL) receptor-deficient mice (LDLr^−/−^/MD-1^+/−^ and LDLr^−/−^/MD-1^−/−^). In contrast to RP105 deficient mice in previous reports, MD-1 deficiency did not clearly influence atherosclerosis development. However, LDLr^−/−^/MD-1^−/−^ mice exhibited significantly higher serum levels of total protein, triglycerides, cholesterol, LC/MS-detected lipophilic compounds, and an increased peripheral B-cell percentage with a Th2 antibody shift after 24 weeks of HFD. Furthermore, lymphocyte infiltration, predominantly of B2B cells and CD4^+^T cells, was visible in random cross-sectional liver sections from LDLr^−/−^/MD-1^−/−^ mice. Our results indicated that MD-1 deficiency leads to further hyperlipidemia, Th2 shift, and enhances lymphocyte infiltration in the LDLr^−/−^ liver.

**Supplementary Information:**

The online version contains supplementary material available at 10.1038/s41598-025-21070-x.

## Introduction

Atherosclerosis is a progressive inflammatory disease characterized by lipid accumulation in the arterial vessel walls^1^. Lipids (cholesterol and cholesterol esters) in the blood enter the arterial wall as lipoproteins from damaged endothelial cells, causing local vessel inflammation. Low-density lipoproteins (LDLs) are one of the five major groups of lipoproteins that transport lipids, such as cholesterol and triglycerides, within the bloodstream^2^. High plasma LDL levels are among the most prominent risk factors for atherosclerosis^1^. LDL receptors recognize ApoB100 and ApoE which are the surface apolipoproteins of LDLs and mediate the uptake of LDL particles through endocytosis. LDL receptors maintain the plasma level of LDLs and are fundamental for the clearance of LDLs from circulation by the liver^3^.

Toll-like receptors (TLRs) are the most characteristic pattern recognition receptors (PRRs) that play vital roles in the innate immunity system. They are major contributors to the development of cardiovascular disease^4,5^. For example, cell surface TLRs, such as TLR1, TLR2, TLR6, and TLR4, contribute to atherosclerosis in LDLr^−/−^ mice^6–8^. Furthermore, mice deficient in TLR4 and its downstream adaptor protein MyD88 show reduced atherosclerosis^9,10^. The mechanisms of atherogenesis induced by TLRs include vascular cell dysfunction, recruitment of macrophages and other immune cells to the site of vascular injury, foam cell formation, and plaque instability^11^.

TLR activation and signaling are regulated by several accessory molecules^12^. TLR4 binds to MD-2, a soluble glycoprotein, and the TLR4/MD-2 complex recognizes lipopolysaccharide (LPS)^13–15^. RP105, which structurally resembles TLR4, binds to MD-1, another soluble glycoprotein similar to MD-2^16^. MD-1 is indispensable for the cell surface expression of RP105^17^, and the RP105/MD-1 complex is expressed on B cells^17,18^, macrophages^18^, and dendritic cells^19^. This complex enhances cell-surface TLR responses in B cells^18^ while negatively regulating TLR4/MD-2 in dendritic cells^19^. RP105 deficiency has been shown to attenuate atherosclerosis^20,21^, with studies indicating reduced atherosclerotic lesion formation in bone marrow chimeras due to alterations in pro-inflammatory B2B cells^20^. Furthermore, RP105-deficient mice exhibit fewer atherosclerotic changes, potentially due to CCR2 downregulation in LDLr^−/−^/RP105^−/−^ monocytes compared with LDLr^−/−^ monocytes^21^.

MD-1 is involved in inflammation, obesity, and insulin resistance^19,22,23^. Previous reports have reported worsened cardiac pathology in MD-1-deficient mice, including pressure overload-induced cardiac remodeling and HFD-induced inflammatory atrial fibrosis^24,25^. Furthermore, *ly86* has been identified as an upregulated gene in atherosclerosis through DEG database analysis^26^, with its expression significantly elevated in human atherosclerotic plaques^27^. However, there are no reports on whether MD-1 affects atherosclerotic changes, and its involvement in the development of atherosclerosis remains unclear.

In this study, we analyzed the effect of MD-1 deficiency on the development of HFD-induced atherosclerosis using littermates of LDL receptor double-deficient mice (LDLr^−/−^/MD-1^+/−^ and LDLr^−/−^/MD-1^−/−^). While no clear differences in atherosclerotic changes were observed between these groups, MD-1 deficiency altered the serum levels of several chemical lipids, immunoglobulins, peripheral B cell percentage, and Th2 shift. Unexpectedly, lymphocyte infiltration in the liver was more visible in LDLr^−/−^/MD-1^−/−^ mice than in LDLr^−/−^/MD-1^+/−^ mice in random cross-sectional liver sections. These results suggest that MD-1-deficiency affects lipid metabolism, lymphocyte character, and lymphocyte infiltration in the liver.

## Results

### Serum levels of total protein, triglyceride, total cholesterol, and LDL cholesterol were higher in LDLr^−/−^/MD-1^−/−^ mice compared with LDLr^−/−^/MD-1^+/−^ mice following HFD feeding

**Fig. 1:**
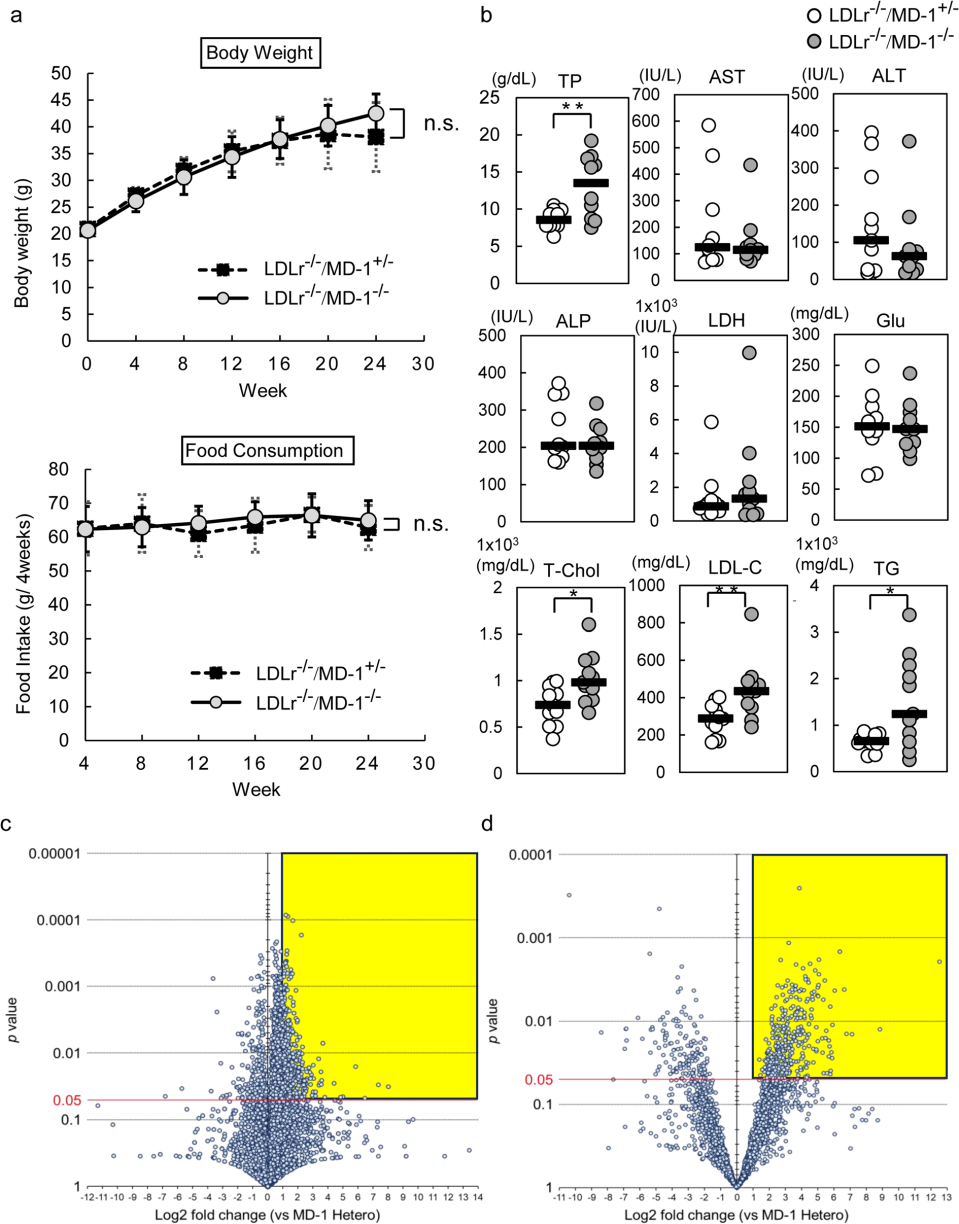
MD-1 deficiency enhances hyperlipidemia. (**a**) (Upper) Changes in body weight and (Lower) food consumption in LDLr^−/−^/MD-1^−/−^ mice and LDLr^−/−^/MD-1^+/−^ mice after 24 weeks of HFD (*n* = 11 per group). Data are presented as the mean ± standard deviation (SD). n.s.,  not significant (Mann–Whitney U test). (**b**) After 24 weeks of HFD, serum levels of total protein, total cholesterol, LDL-cholesterol, and triglycerides were significantly higher in LDLr^−/−^/MD-1^−/−^ mice than LDLr^−/−^/MD-1^+/−^ mice. Data are presented as median (LDLr^−/−^/MD-1^+/−^ mice: *n* = 10 for TP, AST, ALP, and glucose; *n* = 11 for ALT, LDH, T-Cho, LDL-C, and TG; LDLr^−/−^/MD-1^−/−^ mice: *n* = 10 for TP, AST, ALP, and glucose; *n* = 11 for ALT, LDH, T-Cho, LDL-C, and TG). Statistical comparisons were performed using Student’s* t*-test (Glu, T-Chol, LDL-C), Welch’s *t*-test (TP and TG), and Mann–Whitney U test (AST, ALT, ALP, and LDH). *P < 0.05, **P < 0.01. (**c**, **d**) Serum lipid content after 24 weeks of HFD in LDLr^−/−^/MD-1^−/−^ mice, expressed as a ratio compared to LDLr^−/−^/MD-1^+/−^ mice, presented in a volcano plot. (**c**) Positive ion mode; (**d**) Negative ion mode. Statistically significant increased lipids (*P* < 0.05, Log2 fold change > 1) are highlighted in yellow in the upper and right corners (Student’s *t*-test).

A previous study showed that B6/MD-1^−/−^ mice have increased energy expenditure and are protected from HFD-induced obesity and hepatic steatosis, similar to that of B6/RP105^−/−^ mice^22^. To analyze whether this effect was also observed in LDLr^−/−^/MD-1^−/−^ mice, we monitored body weight over time. Contrary to prior findings, LDLr^−/−^/MD-1^−/−^ mice exhibited progressive weight gain, with no significant difference compared to LDLr^−/−^/MD-1^+/−^ mice, although the latter showed a trend toward a plateau (Fig. 1a, upper). Furthermore, we did not observe a clear difference in food intake between the two groups (Fig. 1a, lower panel). However, the results of serum biochemistry tests showed significant differences. Total protein, triglyceride (TG), total cholesterol, and low-density lipoprotein cholesterol (LDL-C) levels were higher in LDLr^−/−^/MD-1^−/−^ mice than in LDLr^−/−^/MD-1^+/−^ mice (Fig. 1b), suggesting a hyperlipidemic tendency in LDLr^−/−^/MD-1^−/−^ mice. We performed a comprehensive analysis using LC/MS to determine whether there were differences in the types and amounts of serum lipids. Lipidomics detects positive and negative ions in the positive and negative modes, respectively. In the positive mode, phosphatidylcholine, sphingomyelin, and triacylglycerol were mainly detected. In the negative mode, phosphatidylethanolamine, NEFAs, and ceramides were mainly detected. In the volcano plot of the positive ion mode, approximately 8,500 lipophilic compound peaks were detected (8496 in LDLr^−/−^/MD-1^+/−^ vs. 8469 in LDLr^−/−^/MD-1^−/−^), and 6154 peaks were elevated in LDLr^−/−^/MD-1^−/−^ mice, with 1901 showing significant increases (Fig. 1c, yellow area). In negative ion mode, approximately 4100 peaks were detected (4137 in LDLr^−/−^/MD-1^+/−^ vs. 4149 in LDLr^−/−^/MD-1^−/−^), and 2642 peaks were higher in LDLr^−/−^/MD-1^−/−^ mice, with 572 showing significant increases (Fig. 1d, yellow area). The shapes of the two volcano plots are different owing to the difference in the number of ions detected; however, there is no characteristic change in the type of lipid in either the positive or negative mode, and both modes show similar trends. Lipids in the area circled in yellow in the upper right of both modes were significantly upregulated compared to LDLr^−/−^/MD-1^+/−^. These lipids are not limited to any particular group, as they are generally accepted regardless of their type. From these results, we concluded that this is consistent with the fact that LDLr^−/−^/MD-1^−/−^ mice have more widely expressed lipids in both ionic modes and total lipids such as serum TG or Cholesterol are elevated in the serological results of LDLr^−/−^/MD-1^−/−^ mice. (Fig. 1b). Previous studies have shown that MD-1 has a hydrophobic cavity to which endogenous phospholipids bind^28^. Our results suggest that lipid binding is less restrictive in the MD-1-deficient state. These results indicate that MD-1 deficiency enhances hyperlipidemia in LDL receptor-deficient mice.

### MD-1 deficiency did not affect atherosclerosis formation

**Fig. 2:**
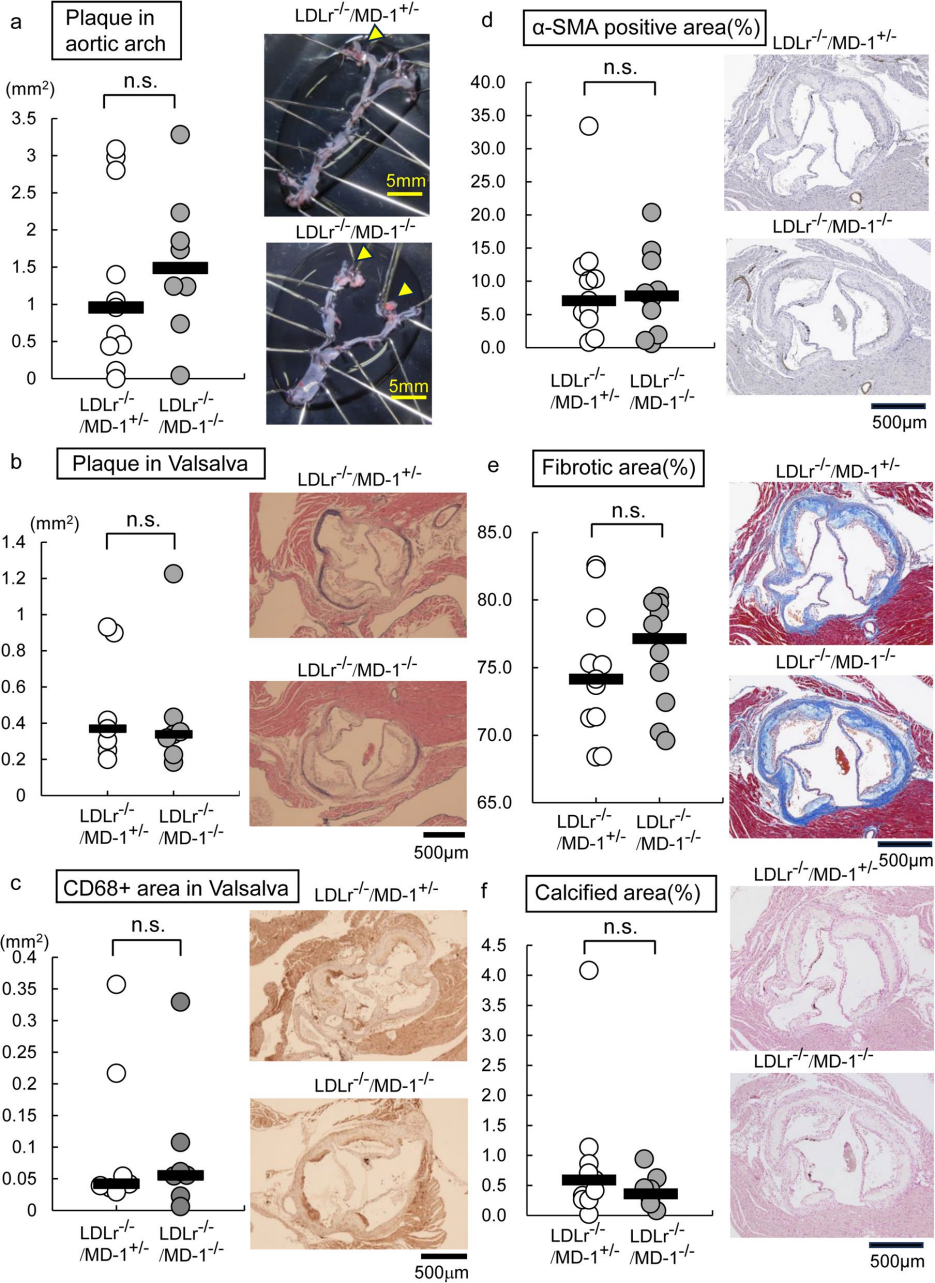
No significant differences in atherosclerotic lesion areas after 24 weeks of HFD were observed between LDLr^−/−^/MD-1^−/−^ and LDLr^−/−^/MD-1^+/−^ mice. (**a**) (Left): Atherosclerotic lesions in the thoracic aortic arch and brachiocephalic artery (LDLr^−/−^/MD-1^+/−^ mice: *n* = 11, LDLr^−/−^/MD-1^−/−^ mice: *n* = 8, Mann–Whitney U test). (Right) Representative en face images of aortic arches with lipid-laden plaques stained with Sudan IV (yellow arrows indicate lipid-laden plaques in the thoracic aorta.). (**b**) (Left) Atherosclerotic lesions at the Valsalva sinus in the aortic root (LDLr^−/−^/MD-1^+/−^ mice: *n* = 7, LDLr^−/−^/MD-1^−/−^ mice: *n* = 7, Mann–Whitney U test). (Right) Representative en face images of aortic roots stained with HE and Victoria blue (purple staining indicates plaque edges.). (**c**) (Left) Macrophage content in the Valsalva sinus in the aortic root (LDLr^−/−^/MD-1^+/−^ mice: *n* = 7, LDLr^−/−^/MD-1^−/−^ mice: *n* = 7, Mann–Whitney U test). (Right) Representative en face images of aortic roots stained with anti-CD68 mAb (brown indicates macrophages). (**d**) (Left) Percentage of α-SMA-positive stained area in the Valsalva sinus (LDLr^−/−^/MD-1^+/−^ mice: *n* = 11, LDLr^−/−^/MD-1^−/−^ mice:* n* = 10, Student’s *t-*test). (Right) Representative en face images of aortic roots stained with α-SMA mAb (bown indicates smooth muscle content). (**e**) (Left) Percentage of Masson’s trichrome positive stained area at the Valsalva sinus (LDLr^−/−^/MD-1^+/−^ mice: *n* = 11, LDLr^−/−^/MD-1^−/−^ mice: *n* = 10, Student *t-*test). (Right) Representative en face images of aortic roots stained with Masson’s trichrome (blue color indicates collagen fibers.). (**f**) (Left) Percentage of von Kossa positive stained area at the Valsalva sinus (LDLr^−/−^/MD-1^+/−^ mice: *n* = 11, LDLr^−/−^/MD-1^−/−^ mice: *n* = 10, Mann–Whitney U test). (Right) Representative en face images of aortic roots stained with von Kossa (black color indicates calcification). Scale bar = a: 5 mm, b-f: 500 μm. All data are presented as median. n.s., not significant.

To assess the role of MD-1 in atherosclerosis, we quantified the atherosclerotic lesions. On lipid-rich plaques in the aortic arch, no statistical difference in the stained lesion size was observed between the two groups of LDLr ^−/−^ mice (median of 0.958 mm^2^ in MD-1^+/−^ mice versus 1.488 mm^2^ in MD-1^−/−^ mice; Fig. 2a). Although we also analyzed plaque size at the Valsalva caves in the aortic root cross-section, the difference was not statistically significant (median of 0.369 mm^2^ in MD-1^+/−^ mice versus 0.338 mm^2^ in MD-1^−/−^ mice; Fig. 2b). A clear difference in the CD68-positive migration area of macrophages was not observed between these two groups (median of 0.042 mm^2^ in MD-1^+/−^ mice versus 0.056 mm^2^ in MD-1^−/−^ mice; Fig. 2c). Further, to analyze the key stability features of atherosclerosis such as fibrous cap area, collagen content, and calcification, we conducted immunohistochemistry of anti-smooth muscle actin antibody (α-SMA) staining, Masson’s trichrome staining, and von Kossa staining. Evaluation of the percentage of stained positive area relative to the cross-sectional area near the Valsalva sinus of the heart also showed no statistical difference between the two groups of LDLr^−/−^ mice (median of 7.06% in MD-1^+/−^ mice versus 7.8085% in MD-1^−/−^ mice; Fig. 2d, median of 74.144% in MD-1^+/−^ mice versus 77.1365% in MD-1^−/−^ mice; Fig. 2e, median of 0.591% in MD-1^+/−^ mice versus 0.3585% in MD-1^−/−^ mice; Fig. 2f). Overall, none of the results were statistically significant, owing to the large individual differences.

### Increase of peripheral B cells and serum IgE levels but decrease of serum IgG2c levels in LDLr^−/−^/MD-1^−/−^ mice

**Fig. 3:**
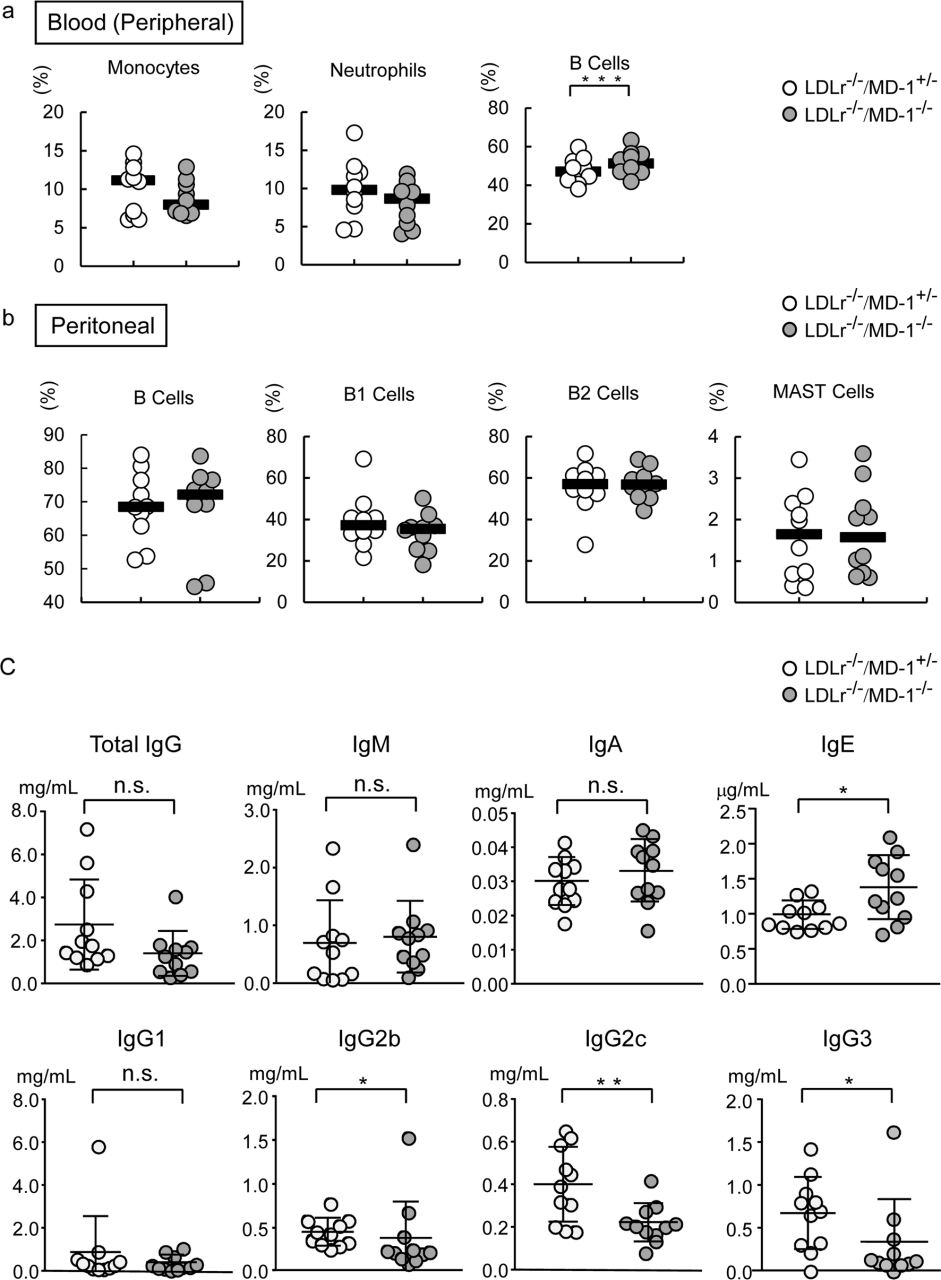
Statistical differences were observed in the peripheral B cell percentage and serum IgG2b, IgG2c, IgG3, and IgE levels of LDLr^−/−^/MD-1^−/−^ mice compared with those of LDLr^−/−^/MD-1^+/−^ mice. (**a**) The percentages of peripheral monocytes, neutrophils, and B cells after 24 weeks of HFD are shown. (LDLr^−/−^/MD-1^+/−^ mice: *n* = 10, LDLr^−/−^/MD-1^−/−^ mice: *n* = 10). Mann–Whitney’s U test (monocytes) and Student’s *t*-test (neutrophils and B Cells) were used to compare two groups. The bar represents the median. (**b**) The percentage of peritoneal B cells, B1 cells, B2 cells, and mast cells after 24 weeks of HFD is shown (LDLr^−/−^/MD-1^+/−^ mice: *n* = 10, LDLr^−/−^/MD-1^−/−^ mice: *n* = 10). Mann–Whitney’s U test (B cells) and Student’s *t*-test (B1 cells, B2 cells, and mast cells) were used to compare two groups. The bar represents the median. ***P < 0.005. (**c**) Serum levels of IgG, IgG1, IgG3, IgG2b, IgG2c, IgA, IgM, and IgE at 24 weeks of HFD were measured using ELISA (LDLr^−/−^/MD-1^+/−^ mice: *n* = 11, LDLr^−/−^/MD-1^−/−^ mice: *n* = 11). The statistical analysis was performed using Student’s *t*-test (IgA and IgM), Welch’s *t*-test (IgG2c and IgE), or Mann–Whitney’s U test (total IgG, IgG1, IgG3, and IgG2b). *P < 0.05, **P < 0.01. n.s., not significant.

**Fig. 4:**
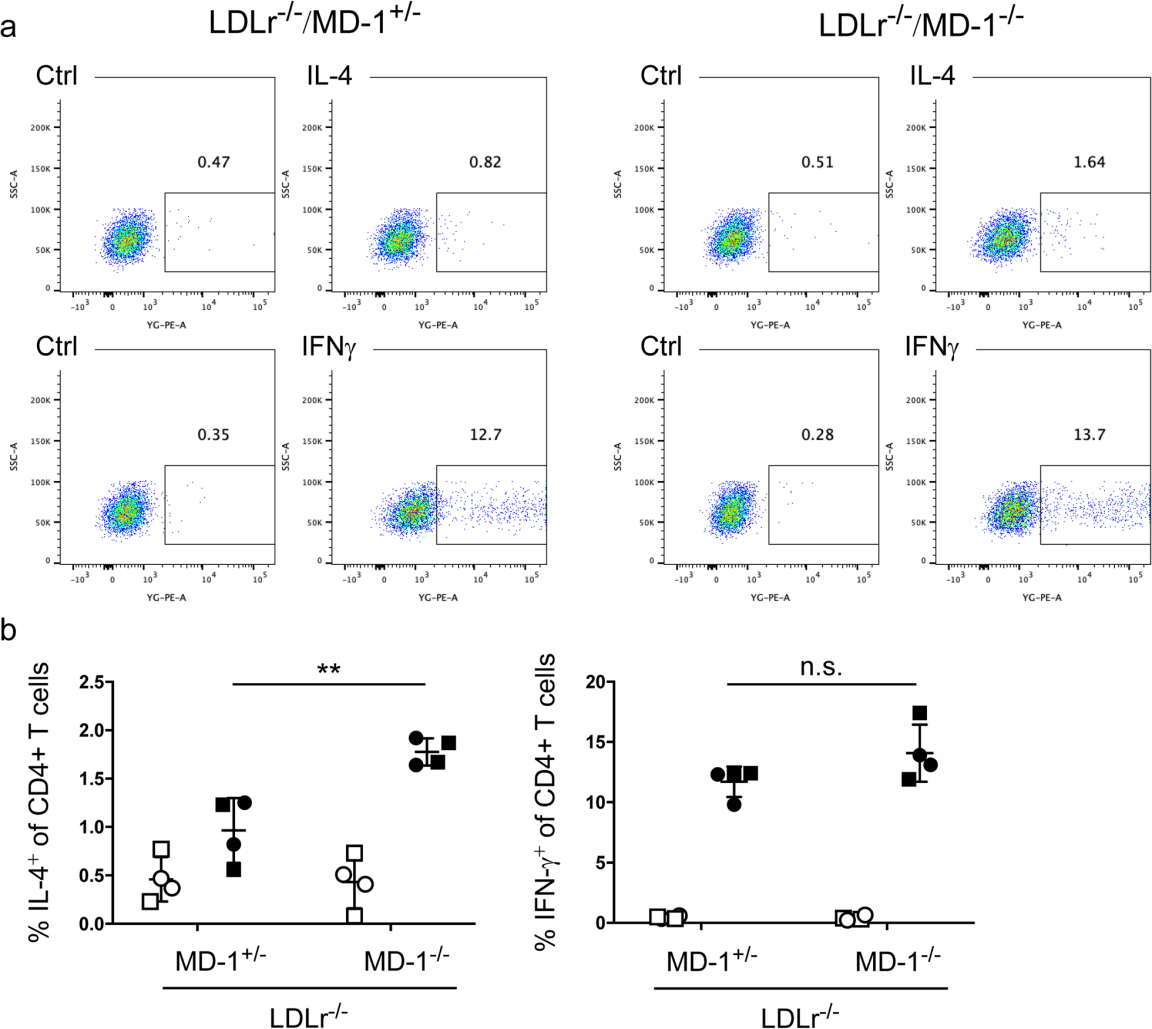
LDLr^−/−^/MD-1^−/−^ mice exhibited an increased frequency of IL-4-producing T cells. (**a**) Flow cytometric analysis of intracellular IL-4 (upper panels) and IFN-γ (lower panels) in splenic CD4^ +^ T cells from LDLr^−/−^/MD-1^+/−^ and LDLr^−/−^/MD-1^−/−^ mice. Splenocytes were stimulated with PMA and ionomycin in the presence of brefeldin A for 4 h. (**b**) Frequency of IL-4^+^ (left panel) and IFN-γ^+^ (right panel) cells in CD4^+^ T cells shown in (**a**). Data are mean ± SD of four independent samples (2 males shown in circles and 2 females shown in squares). Open symbols indicate isotype-matched control mAb staining and closed symbols indicate anti-IL-4 mAb staining (left panel) or anti- IFN-γ mAb staining (right panel). **P < 0.01, Statistical differences are determined by Student’s *t*-test. n.s., not significant.

As MD-1 deficiency affects B cell activation^17,18^, we further analyzed the percentage of B cells in the peripheral blood and peritoneum using fluorescence-activated cell sorting (FACS). The percentage of B cells (B220^+^CD3^−^ cells; Supplementary Fig. 1, lower) in the peripheral blood was significantly increased in MD-1 deficient mice (median of LDLr^−/−^/MD-1^+/−^ mice: 47.05% vs. LDLr^−/−^/MD-1^−/−^ mice: 51.25%; *P* < 0.005; Fig. 3a). Confirmation of MD-1 expression in the peripheral blood cells of LDLr^−/−^/MD-1^+/−^ and LDLr^−/−^/MD-1^−/−^ mice is shown in Supplementary Fig. 2. The peritoneal B cell percentage (CD19^+^ cells; Supplementary Fig. 3, middle), including B cell subset content (B1 cells (CD19^+^CD23^-^cells) and B2 cells (CD19^+^CD23^+^CD5^-^cells); Supplementary Fig. 3, lower), was not statistically different between these groups (Fig. 3b). In addition, mast cell content did not differ between the two groups (Fig. 3b; Supplementary Fig. 4). Peripheral neutrophils tended to decrease in LDLr^−/−^/MD-1^−/−^ mice compared to LDLr^−/−^/MD-1^+/−^ mice, but the difference was not statistically significant (Fig. 3a; Supplementary Fig. 1, middle). In summary, LDLr^−/−^/MD-1^−/−^ mice tended to have a higher percentage of peripheral blood B cells. Next, we analyzed the serum antibody titers. A previous report showed that MD-1 deficiency reduces IgG2b and IgG3 levels, similar to that of RP105 deficiency^29^. In the present study, the same reduction was observed in LDLr^−/−^/MD-1^−/−^ mice. In contrast, serum antibody analysis revealed a statistically significant reduction in IgG2c levels, whereas IgE levels showed a slight increase in LDLr^−/−^/MD-1^−/−^ mice compared with LDLr^−/−^/MD-1^+/−^ mice (Fig. 3c). This result indicates a Th2 shift in antibody production in LDLr^−/−^/MD-1^−/−^ mice. To confirm a Th1/Th2 response shift in LDLr^−/−^/MD-1^−/−^ mice, we conducted FACS analysis to measure IFN-γ and IL-4 levels in splenic lymphocytes (Fig. 4). Despite the normal chow diet, after stimulation with PMA and ionomycin, splenocytes derived from LDLr^−/−^/MD-1^−/−^ mice showed a statistically significant increase in IL-4-producing T cells compared to cells derived from LDLr^−/−^/MD-1^+/−^ mice, indicating a shift toward Th2.

### MD-1 deficiency did not affect the lipid accumulation in liver and the statistical hepatic score

**Fig. 5:**
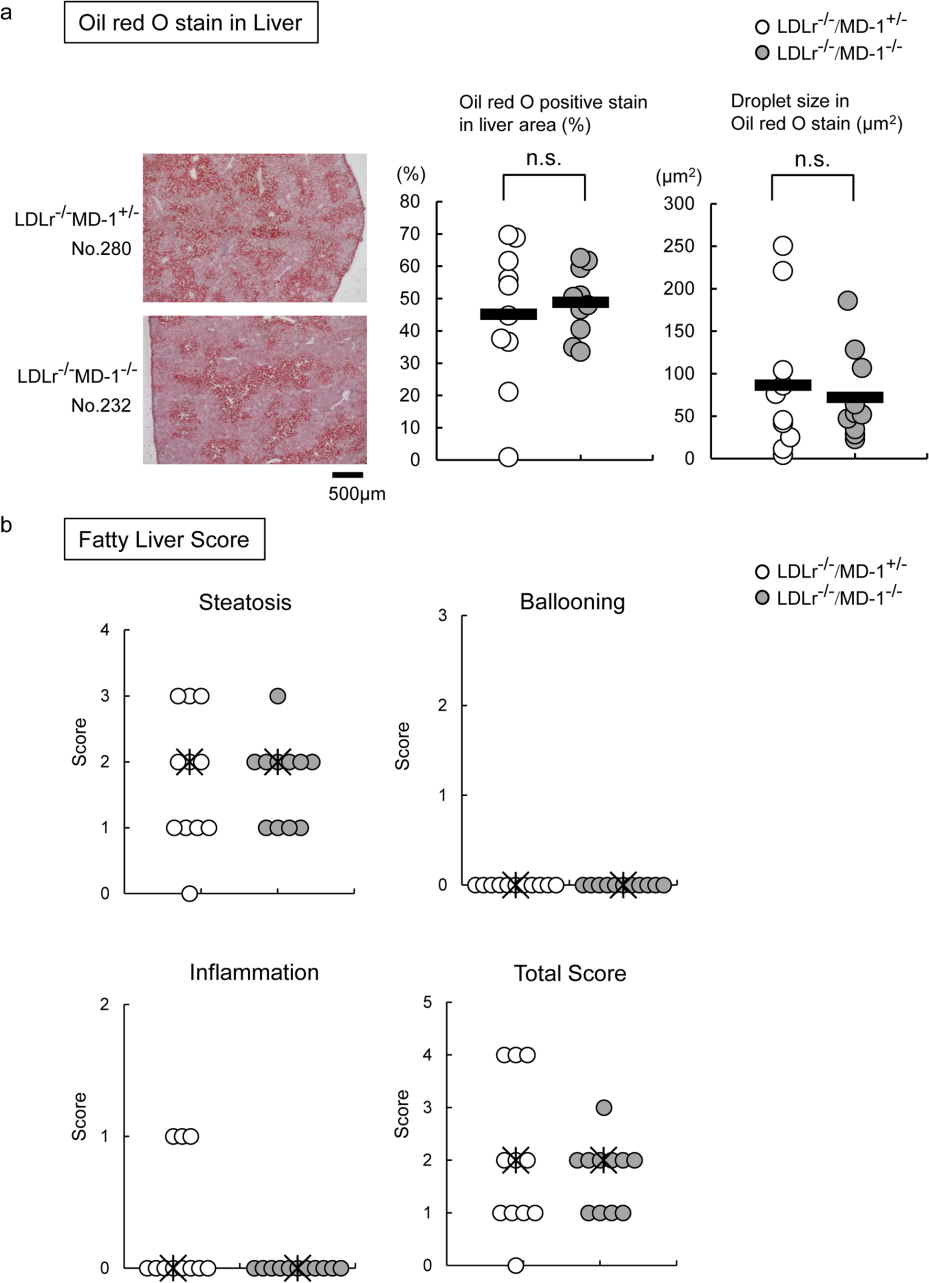
The statistical difference of lipid accumulation and that of hepatocellular injury was not observed between LDLr^−/−^MD-1^−/−^ mice and LDLr^−/−^/MD-1^+/−^ mice. (**a**) Left: Representative photographs of Oil Red O stain of frozen liver sections are shown. Middle: The percentage of positively stained areas relative to liver areas (LDLr^−/−^/MD-1^+/−^ mice: *n* = 10, LDLr^−/−^/MD-1^−/−^ mice: *n* = 10, Welch’s *t*-test). Right: Droplet size in Oil Red O stain. (LDLr^−/−^/MD-1^+/−^ mice: *n* = 10, LDLr^−/−^/MD-1^−/−^ mice: *n* = 10, Welch’s *t*-test test). The bar represents the mean. n.s., not significant. (**b**) Steatosis, ballooning, inflammation, and total scores are shown. (LDLr^−/−^/MD-1^+/−^ mice: *n* = 11, LDLr^−/−^/MD-1^−/−^ mice: *n* = 11, Mann–Whitney’s U test). The asterisk mark denotes the median.

To evaluate local lipid accumulation, we performed Oil Red O staining of frozen liver sections. Contrary to our expectations from the serum chemistry results (Fig. 1b and 1c), no statistical difference was observed between LDLr^−/−^/MD-1^+/−^ and LDLr^−/−^/MD-1^−/−^ mice (Fig. 5a). Further, we examined the Nonalcoholic Fatty Liver Disease (NAFLD) activity score (NAS) in the liver tissues (the score ranges are shown in the supplementary table). Steatosis was also observed, but ballooning was not observed in either LDLr^−/−^/MD-1^+/−^ or LDLr^−/−^/MD-1^−/−^ mice (Fig. 5b). Contrary to the hyperlipidemia tendency, inflammation was recognized in only three LDLr^−/−^/MD-1^+/−^ mice. Hepatocellular injury was rarely observed in either group.

### Lymphocyte infiltration into the liver was elevated in LDLr^−/−^/MD-1^−/−^ mice

**Fig. 6:**
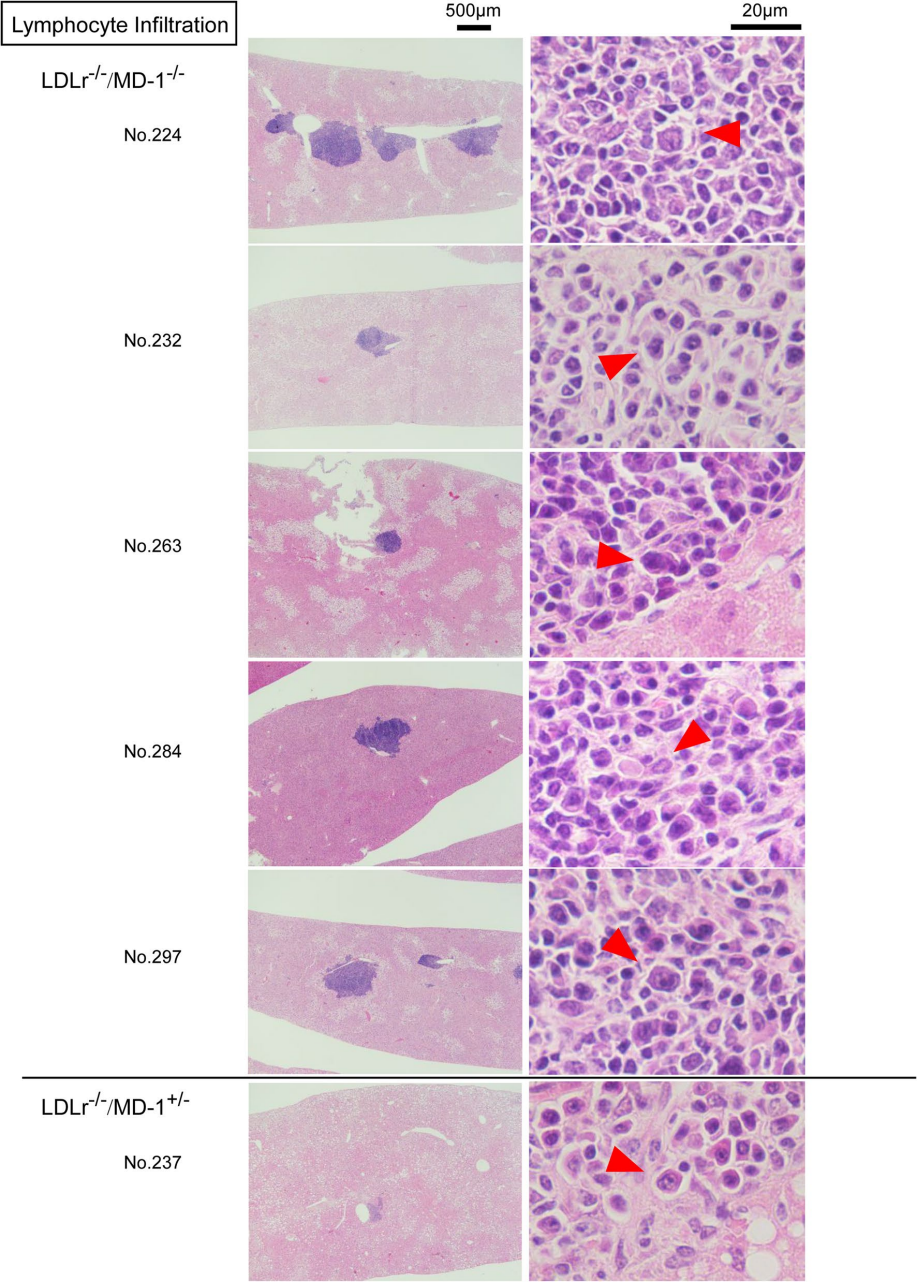
Lymphocyte infiltration was observed in the livers of LDLr^−/−^/MD-1^−/−^ mice and LDLr^−/−^/MD-1^+/−^ mice. Out of 11 mice in each group, 5 LDLr^−/−^/MD-1^−/−^ (No. 224, 232, 263, 284, and 297) and 1 LDLr^−/−^/MD-1^+/−^ mouse (No. 237) showed lymphocyte infiltration in HE-stained liver sections. Left: Low-magnification image (scale bar = 500 μm). Right: High-magnification image (scale bar = 20 µm). The red arrows point to plasma-like cells.

**Fig. 7:**
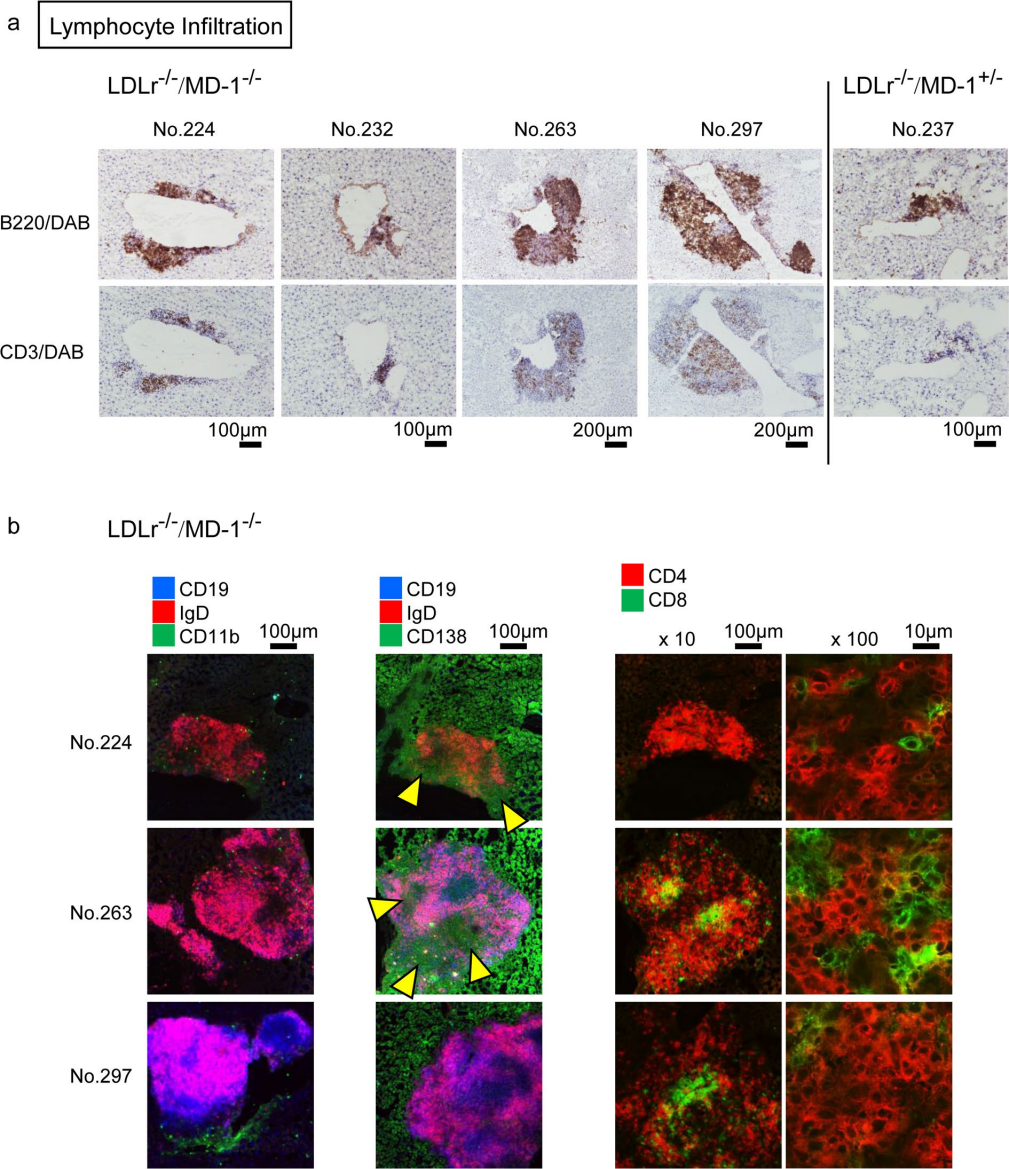
B-cell-driven lymphocyte infiltration was observed in the liver sections of LDLr^−/−^/MD-1^−/−^ mice and one LDLr^−/−^/MD-1^+/−^ mouse. Among the mice livers with lymphocytic infiltration, 4 LDLr^−/−^/MD-1^−/−^ (No. 224, 232, 263, and 297) and 1 LDLr^−/−^/MD-1^+/−^ (No. 237) mouse exhibited clear lymphocyte infiltration even in the liver frozen sections. (**a**) Upper: B220/DAB staining. Lower: CD3/DAB staining. Lymphocyte infiltration was not observed in frozen liver sections from No. 284, which were derived from consecutive cuts at various angles from the remaining liver block. Scale bars: 100, or 200 µm. (**b**) Confocal analysis image of multiple fluorescence staining. Left: CD19/IgD/CD11b staining. Middle: CD19/IgD/CD138 staining. Right: CD4/CD8 staining. Scale bars: 100, or 10 µm.

Despite the absence of significant inflammation or hepatocellular injury, lymphocytic infiltration was unexpectedly observed, mainly along the portal vein of the liver tissue. Liver cross-sections showed visible lymphocyte infiltration in 5 of 11 LDLr^−/−^/MD-1^−/−^mice, compared to only 1 of 11 LDLr^−/−^/MD-1^+/−^ mice (Fig. 6). These lymphocytes included the area of increased plasma cell-like cells with large cytoplasms (red marks indicates a typical plasma cell-like cells among them in the right image in Fig. 6).Although no statistical significance was observed in the percentage of infiltrated areas relative to the total liver area, the percentages of 5 LDLr^−/−^/MD-1^−/−^ mice were higher than those of the other mice (Supplementary Fig. 5a). Even in the normal chow diet, the infiltration was also recognized, but the average percentage of the infiltrated area was lower than that of 24 weeks HFD-treated LDLr^−/−^/MD-1^−/−^ mice (Supplementary Fig. 5b). This result suggests that HFD enhanced lymphocyte infiltration in liver. Immunohistochemical staining revealed that most infiltrating lymphocytes were B cells, as indicated by an increased B220-positive area compared to the CD3-positive area in the frozen sections (Fig. 7a). To confirm the subpopulation of infiltrated lymphocytes in the liver, we stained frozen sections with antibodies against lymphocyte subsets. Of the five samples from which frozen sections were prepared, antibody-positive lymphocyte infiltration was detected in three samples (Fig. 7b). In mouse No. 224, IgD^+^ single-positive cells mainly increased, whereas in mice No. 263 and No. 297, IgD^+^CD19^+^ dual-positive B2 cells mainly increased. CD11b^+^B1 cells were rarely observed in any sample (Fig. 7b, left). CD138 is expressed on the surface of pre-B cells and plasma cells, but also expressed in epithelium, fibroblasts, vascular smooth muscle cells, and endothelial cells^30^. Mainly increased cells were CD138^+^IgD^+^ cells in No. 224, and CD138^+^IgD^+^CD19^+^ triple positive cells in No. 263, these cells may be different from plasma cells, such as pre-plasma cells^31^. Whereas we were able to find the area of CD138^+^IgD⁻CD19⁻ plasma cells (indicated as yellow marks) in both samples, although in No. 297, the cells were rarely observed (Fig. 7b, middle). These results show that the infiltrated B cells were primarily B2 cells, and CD138^+^ plasma cells were also recognized, which was consistent with the data shown in Fig. 6. CD8^+^T cells were recognized; however, CD4^+^ T cells were predominant in all samples (Fig. 7b, right). CD4^+^ T cells belong to the helper T cell group, and Th2 cells, which are also involved in antibody production, are included in this group. This result may be related to the Th2 shift in LDLr^−/−^/MD-1^−/−^ mice (Fig. 4).

### Lymphocyte infiltration correlates with the local lipid accumulation in total LDLr⁻^/^⁻ mice liver

**Fig. 8:**
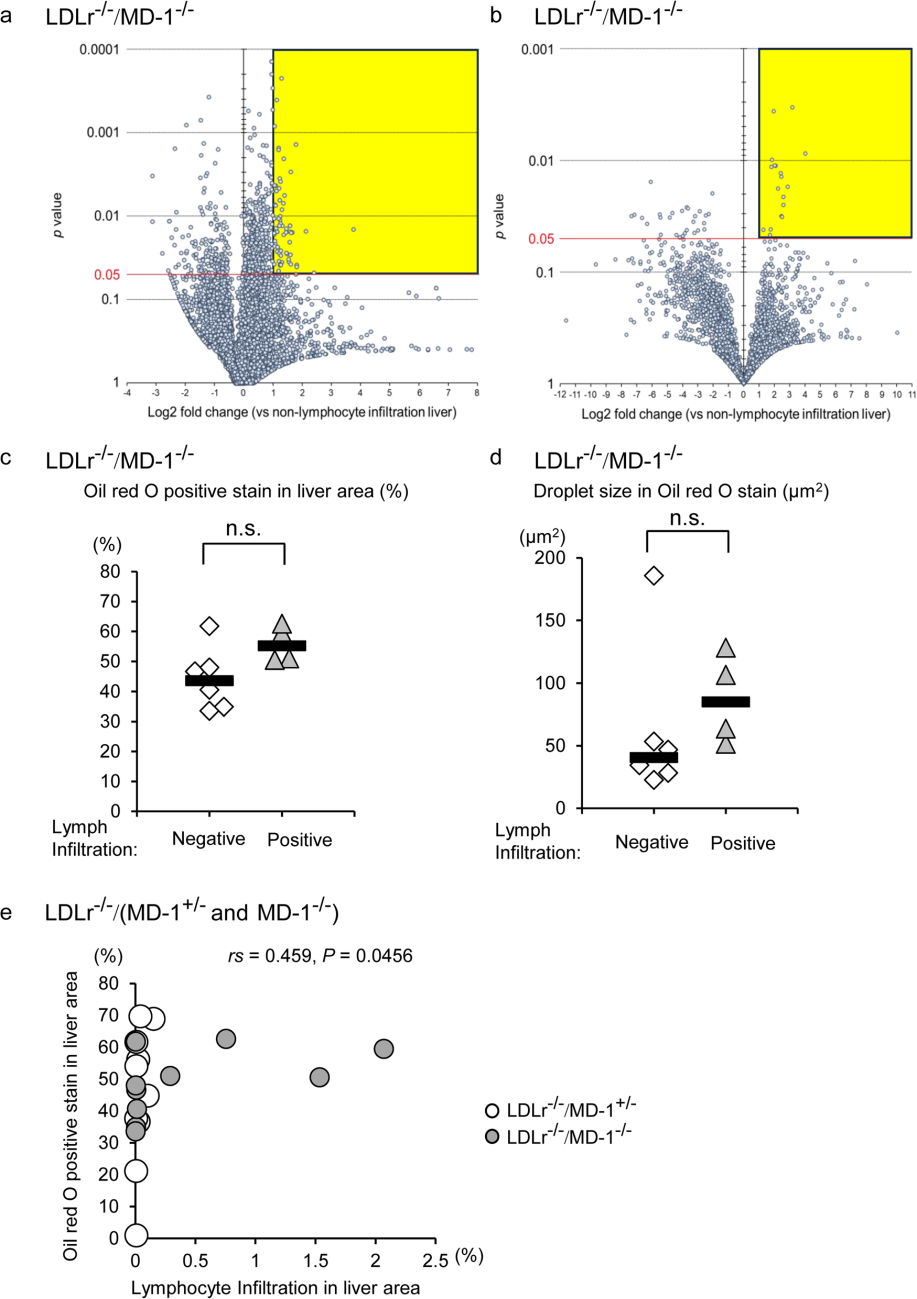
The percentage of the area of lymphocyte infiltration correlates with that of the local lipid accumulation in LDLr⁻/⁻ mice liver. After 24 weeks of HFD, the serum lipid levels in LDLr^−/−^/MD-1^−/−^ mice with liver lymphocyte infiltration were plotted relative to those in LDLr^−/−^/MD-1^−/−^ mice without infiltration, presented as a volcano plot. (**a**): Positive ion mode. (**b**): Negative ion mode. The plots in the upper (*P* < 0.05) and right (Log2 fold change > 1) corners (yellow area) indicate lipids that were significantly increased in LDLr^−/−^/MD-1^−/−^ mice with liver lymphocyte infiltration compared to those without infiltration (Student’s *t*-test). Comparison of (**c**) the percentage of Oil red O positive stained areas relative to total liver areas, or (**d**) the droplet size of Oil red O positive stained in the liver in LDLr^−/−^/MD-1^−/−^ mice between lymphocyte infiltration negative and positive groups (negative: *n* = 6, positive: *n* = 4, (**c**): Student’s *t*-test, (**d**): Mann–Whitney’s U test, These results are modified based on the results of the LDLr^−/−^/MD-1^−/−^ mice in Fig. 5a.). The bar represents the median. n.s., not significant. (**e**) Scatter plot showing the correlation between the percentage of Oil red O positive staining and that of the positive area of lymphocyte infiltration in all (LDLr^−/−^/MD-1^+/−^ and LDLr^−/−^/MD-1^−/−^) mice. White circle: LDLr^−/−^/MD-1^+/−^ mice. Grey circle: LDLr^−/−^/MD-1^−/−^ mice. The correlation coefficient was 0.459 (*P* = 0.0456), suggesting a significant correlation between these two variables. (LDLr^−/−^/MD-1^+/−^ mice: *n* = 10, LDLr^−/−^/MD-1^−/−^ mice: *n* = 10, Spearman’s correlation coefficient by rank test. These results are modified based on the results of all mice in Fig. 5a and Supplementary Fig. 5a.). *P < 0.05.

We investigated whether lipid levels increased in lymphocyte-infiltrated mice using LC/MS analysis. Among the detected lipophilic compound peaks in the positive ion mode (8468 peaks from infiltrated lymphocytes in the liver of LDLr^−/−^/MD-1^−/−^ mice vs. 8347 peaks from lymphocyte non-infiltrated LDLr^−/−^/MD-1^−/−^ mice), 3675 peaks were higher in lymphocyte-infiltrated mice, of which only 661 peaks were significantly increased in lymphocyte-infiltrated mice; however, these increased lipophilic compounds showed only a sporadic increase and could not be interpreted as a meaningful increase (Fig. 8a, plot of the area encircled in yellow). Among the detected peaks in the negative ion mode (4007 peaks in lymphocyte-infiltrated livers vs. 4112 in non-infiltrated livers of LDLr^−/−^/MD-1^−/−^ mice), 1829 peaks were elevated in infiltrated livers, with only 58 showing significant increases. However, these elevated lipophilic compounds appeared sporadically and did not form distinct lipid profiles (Fig. 8b, yellow-circled area). Further, there is no statistical correlation between the serum level of TG, T-Chol, or LDL-C and the percentage of lymphocyte infiltration in the liver (Data not shown). These findings suggest that serum hyperlipidemia in LDLr^−/−^/MD-1^−/−^ mice are not directly associated with lymphocyte infiltration in the liver.

Next, we analyzed the relationship between local lipid accumulation and lymphocyte infiltration in the liver. In LDLr^−/−^/MD-1^−/−^ mice, there was no statistical difference in the percentage of Oil red O positive stained areas relative to the total liver area, or the droplet size of Oil red O positive stain between the lymphocyte infiltration-negative and positive groups (Fig. 8c and 8d). In contrast, in all mice (both LDLr^−/−^/MD-1^**+/−**^ and LDLr^−/−^/MD-1^−/−^), the scatter plot shows the correlation between the percentage of Oil red O positive staining and lymphocyte infiltration. The correlation coefficient was 0.459 (*P* = 0.0456), suggesting a significant correlation between these two variables (Fig. 8d). The correlation was not clear in LDLr^−/−^/MD-1^−/−^ mice only, probably due to the small sample size.

## Discussion

In this study, we demonstrated that MD-1 deficiency augmented HFD-induced hyperlipidemia in LDLr^−/−^ mice. To the best of our knowledge, this is the first report of HFD in LDL and MD-1 double-knockout mice. Previous reports using B6/MD-1^−/−^ mice showed similar but opposite results. For example, Shen et al. reported that 20 weeks of HFD feeding induced statistically high levels of total cholesterol and triglyceride in B6/MD-1^−/−^ mice compared with age-matched B6 wild-type mice^25,32^. In contrast, Watanabe et al. reported that serum cholesterol and glucose levels were lower in B6/MD-1^−/−^ mice than in B6 wild-type mice fed HFD for 12 weeks ^22^. This discrepancy may stem from differences in experimental conditions, including HFD duration and mouse strain background (e.g., B6 LDLr^−/−^/MD-1^−/−^ vs. B6/MD-1^−/−^). A strong association between MD-1 and obesity has been shown in several other studies, including RNA expression profile datasets and genome-wide association studies (GWAS). *Ly86* is a promising biomarker for obesity and periodontitis^33^. Furthermore, elevated *ly86* methylation has been observed in obese patients across several genome-wide methylation panels, and *ly86* methylation is associated with insulin resistance and inflammatory markers^23^. These findings underscore the role of MD-1 in obesity-related metabolic disorders. Our results are consistent with these reports.

RP105 or MD-1 deficiency reduces serum IgG2b and IgG3 production^29^, particularly IgG3, which depends on RP105/MD-1 signaling. B cell activation via TLR2/4 and RP105 is indicated by γ3 germline transcript expression and serum IgG3 production, both of which are impaired in the absence of RP105 or TLR2/4 expression. In this study, LDLr^−/−^/MD-1^−/−^ mice exhibited a similar trend. Notably, a Th2-dominant response in MD-1-deficient mice, characterized by slightly elevated IgE and clearly reduced IgG2c levels was found in this study (Fig. 3c). Previously, severe hypercholesterolemia induced a strong Th2 response to antigen-specific antibody production in ApoE-deficient mice, with a correlation between plasma cholesterol levels and the Th2/Th1 ratio^34^. Hence, HFD- LDLr^−/−^/MD-1^−/−^ mice were associated with this switch to Th2. Indeed, Th2 polarization was recognized in the LDLr^−/−^/MD-1^−/−^ mice relative to LDLr^−/−^/MD-1^**+/−**^ mice even on normal chow diet (Fig. 4). This means that MD-1 existence suppressed the Th2 shift in LDLr^−/−^mice.

RP105 deficiency attenuates atherosclerosis by decreasing monocyte influx. Karper et al. showed that RP105^−/−^ bone marrow-transferred LDLr^−/−^ mice displayed reduced plaque burden, and activated B cells and lesional macrophages decreased compared with wild-type transferred LDLr^−/−^ mice^20^. Similarly, Wezel et al. reported that RP105 deficiency reduced plaque size and decreased lesional macrophages, peritoneal B cells, and plasma levels of IgE and IgM in LDLr^−/−^ and LDLr^−/−^/RP105^−/−^ mice^21^. Despite variations in experimental approaches, these findings suggest that RP105 deletion mitigates atherosclerosis. However, we did not observe a clear reduction in atherosclerosis in LDLr^−/−^/MD-1^−/−^ mice. These results suggest that the phenotypes of MD-1 and RP105 deficiencies differ in atherosclerosis.

Coincidentally, lymphocyte infiltration into the liver was more pronounced in LDLr^−/−^ /MD-1^−/−^ mice than in LDLr^−/−^/MD-1^**+/−**^ mice. However, among LDLr^−/−^/MD-1^−/−^ mice, serum lipid profiles did not differ significantly between those with and without lymphocyte infiltration (Fig. 8a and 8b). In contrast, positive correlation between local lipid accumulation and lymphocyte infiltration in the livers of total (both LDLr^−/−^/MD-1^+/−^ and LDLr^−/−^/MD-1^−/−^) mice was observed (Fig. 8e). Probably due to the small sample size, a statistical correlation was not observed only in LDLr^−/−^/MD-1^−/−^ (Fig. 8c and 8d). Infiltrating lymphocytes are predominantly B cells mainly B2 cells; however, they also contain plasma cells, which are characteristic of autoimmune hepatitis in humans^35^. However, there was no evidence of liver damage or systemic inflammation, as the serum levels of IL-1β, TNF-α, and IL-6 in LDLr^−/−^ MD-1^−/−^ mice remained unchanged (data not shown). Therefore, lymphocyte infiltration in these mice appeared to be unrelated to hepatitis.

Recently, lymphocyte infiltration in the liver, particularly in cases of obesity or NAFLD, has been reported. HFD increased liver B2B cells and serum IgG2c production in B6 mice^36^. In other papers, it showed that the level of CD4^+^T, CD8^+^ T, and B cell migration to the liver was significantly higher for HFD lymphocytes than for controls, by using the lymphocyte transfer experiments to LPS-challenged lean recipient mice^37^. Further, adaptive transfer model indicated that mesenteric lymph nodes cells from NAFLD donor mice predominantly accumulated in the livers and induced CD4^+^T and CD8^+^T cells activation and liver injury^38^. In this case, the frequency of Th1 cell (CD4^+^IFNγ^+^T cells) and that of Th17 cell (CD4^+^IL-17^+^T cells) in the liver were increased. Compared to these previous reports, our results showed that the types of cells increasing in the liver were similar, while they differed in exhibiting elevated levels of IgE and Th2 cells (CD4^+^IL-4^+^T cells) compared to controls. In mouse models, NAFLD is characterized by maturation of B2 lymphocytes to plasma cells and by an elevated circulating IgG^36^. B2 cell-derived pathogenic antibodies such as IgG2c have been shown to induce an accumulation of M1 macrophages and Th1 cells^36^.Based on these reports, although the degree of obesity in LDL^−/−^ mice may have been milder compared to other experimental models, the Th2 shift caused by MD-1 deficiency may also have mitigated liver damage due to fatty liver, and, consequently, atherosclerosis with M1 macrophage activation by Th1 shift, even in the hypercholesterolemia.

This study had some limitations. First, we did not identify the mechanism underlying the increased hyperlipidemia in LDLr^−/−^/MD-1^−/−^ mice compared to LDLr^−/−^/MD-1^**+/−**^ mice. Jiang et al. reported that MD-1 promotes ox-LDL-induced lipid accumulation in macrophages by upregulating SREBP2/HMGCR expression^27^; however, this role of MD-1 contradicts our in vivo results. Further analyses are required to elucidate the role of MD-1 in hyperlipidemia. Secondly, we used LDLr^−/−^ mice in this study because of reference with previous reports of LDLr^−/−^/RP105^−/−^ mice, if we changed the more severe mouse model to such as Apo-E^−/−^ may show improvement the atherosclerotic change. Thirdly, we did not identify the reason for the Th2 shift in LDLr^−/−^/MD-1^−/−^ mice. Also, the correlation between hyperlipidemia and Th2 shift was not observed (Data were not shown), although the relation has been reported in Apo-E^−/−^mice. And finally, the use of random cross-sectional liver sections limits the generalizability of our findings. Therefore, more detailed analytical methods are required to determine the degree of lymphocytic infiltration.

In conclusion, MD-1 deficiency affects serum biochemistry, peripheral B cell percentages, serum immunoglobulin levels, Th2 shift, and lymphocyte migration to the liver under HFD conditions. MD-1 deficiency exacerbated hyperlipidemia, and HFD possibly enhances lymphocyte infiltration through local lipid accumulation in LDLr^−/−^ mice liver, although the statistical difference was not observed in only LDLr^−/−^/MD-1^−/−^ mice, probably due to the small sample size. Although further investigation is needed, MD-1 may represent a new target for treating hyperlipidemia and lymphocyte abnormalities.

## Methods

### Animals

B6/LDLr^−/−^ male mice were purchased from the Jackson Laboratory. B6/MD-1^−/−^ female mice were kindly provided by Dr. Kensuke Miyake (Tokyo University, Tokyo, Japan). MD-1 deficient mice were produced as described previously^17^. B6/LDLr^+/−^MD-1^+/−^ mice (F1 mice) were established and mated to generate B6/LDLr^−/−^/MD-1^+/−^ and B6/LDLr^−/−^/MD-1^−/−^ mice. Each LDLr^−/−^/MD-1^+/−^ and LDLr^−/−^/MD-1^−/−^ male mouse pair were full siblings from the same parents. At seven weeks of age, the mice were individually fed HFD (60 kcal% fat, with lard; D12492, Research Diets, New Brunswick, NJ, USA) for the duration of the experiment. Body weights were measured monthly. Food intake was measured every 3 d, totaled for 1 month, and plotted on a graph to show the trend. After 24 weeks of HFD feeding and serum collection, the mice were euthanized using CO2 gas. A portion of the collected serum was used for peripheral blood mononuclear cell extraction using BD PharmLyse™ Lysing Buffer (BD Biosciences, Franklin Lakes, NJ, USA). The remaining portion was used to measure biochemical parameters at Nagahama LSL (Nagahama, Japan), lipid contents, and antibodies.

All animal experiments were performed after obtaining approval from the Animal Research Committee of Aichi Medical University and following the institutional ethical guidelines for animal experiments at Aichi Medical University. The institutional guidelines complied with the ARRIVE guidelines.

### Lipid extraction

For lipid analysis, 20 µL of mouse serum was mixed with 180 µL ultrapure water (FUJIFILM Wako, Osaka, Japan), 200 µL methanol (FUJIFILM Wako), 400 µL dichloromethane (FUJIFILM Wako), and 20 µL of an internal standard solution mixture (10 µL mouse SPLASH LIPIDOMIX mass spec internal standard [Avanti Polar Lipids] and 10 µL 100 µM ^13^C_16_-palmitic acid [Cambridge Isotope Laboratories] in acetonitrile). The resulting mixture was agitated vigorously for 1 min, centrifuged at 20,000 × *g* for 10 min, and the organic phase was collected. The remaining precipitate and aqueous phase were mixed with 400 µL of dichloromethane, and the resulting mixture was centrifuged at 20,000 × *g* for 5 min to obtain the organic phase. The combined organic phases were evaporated to dryness in vacuo to yield the corresponding derivatized residues. The residues were dissolved in 100 µL of a mixture of isopropyl alcohol/acetonitrile/water (2:1:1, v/v/v), and 10 µL of each sample was injected into the LC/MS system.

### Liquid chromatography quadrupole time-of-flight mass spectrometry (LC-qTOF/MS/MS)

LC-qTOF/MS/MS analyses were performed using a Shimadzu UHPLC Nexera X2 system (Shimazu, Kyoto, Japan) with a TSKgel ODS-120H column (1.9 μm, 100 mm × 2.0 mm; TOSOH, Tokyo, Japan) and a ZenoTOF 7600 system (SCIEX, Toronto, Canada) with an electrospray ionization device operating in the positive and negative ionization modes. The autosampler injection volume was set to 10 µL, with an eluent flow rate of 0.4 mL/min. Mobile phase A consisted of a 0.1% (v/v) solution of formic acid and 10 mM ammonium formate in acetonitrile/water (60/40, v/v), whereas mobile phase B consisted of a 0.1% (v/v) solution of formic acid and 10 mM ammonium formate in isopropyl alcohol/acetonitrile (90/10, v/v). The linear gradient conditions were as follows: 40% B at 0 min, 43% B at 3 min, 55% B at 15 min, 99% B at 25 min, 99% B at 27 min, and 40% B at 27.01 min, followed by a 2.99 min equilibration time. The detector conditions were as follows: ion spray voltage of 5500 V (positive ion mode) and − 4500 V (negative ion mode), source temperature of 350 °C, ion source gas 1 at 60 psi, ion source gas 2 at 60 psi, declustering potential of 80 V (positive ion mode) and − 80 V (negative ion mode), collision energy of 45 V (positive ion mode) and − 45 V (negative ion mode), and a collision energy spread of 15 V. Nitrogen was used as the collision gas. The raw data “wiff” was used to convert to “abf” format using the ABF converter. Raw data analysis was performed using the MS-DIAL software.

### Assessing atherosclerosis in LDLr^−/−^/MD-1^−/−^ and LDLr^−/−^/MD-1^+/−^ mice

After 24 weeks of HFD feeding, serum was collected from the mice. Mice were euthanized using CO2 gas, and peritoneal cells were collected by flushing the peritoneal cavity with 12 ml RPMI. The mice were then opened, and the right auricle was pre-incised for perfusate drainage and washed by flushing with 10 ml of phosphate-buffered saline (PBS) in a cranial direction from the apex of the heart. The livers were retrieved and divided into two sections. One part was kept frozen, and the other was fixed with 4% paraformaldehyde for hematoxylin and eosin (HE) staining. Furthermore, we performed aortic microdissection according to a previously reported protocol^3^. After removing the trachea, esophagus, lungs, heart, and aorta were collected via microdissection under an SMZ745T stereomicroscope (Nikon, Tokyo, Japan) and fixed with 4% paraformaldehyde for histological and morphometric analyses.

### Histology and morphometry

Paraffin-embedded heart tissue Sections (5-μm thickness) were obtained and serial sections were distributed between consecutive slides. Sections containing the Valsalva cave were used for the plaque analysis. Formalin-fixed, paraffin-embedded heart tissue sections were stained with HE or Victoria blue using standard techniques. Immunohistochemical staining for macrophages (FA-11 [CD68]; Novus Biologicals, Centennial, CO, USA) was performed, followed by incubation with goat anti-rat IgG-HRP (Southern Biotech, Birmingham, AL, USA). According to a previously described procedure^3^, the prepared slides were analyzed using ECLIPSE80i with NIS-Elements D3.1 software (Nikon), and each positively stained area was quantified using the ImageJ software (NIH, Bethesda, MD, USA). Formalin-fixed, paraffin-embedded liver tissue sections were stained with HE and analyzed using a BX51 microscope (Olympus, Tokyo, Japan). The fatty liver score based on HE-stained slides was estimated by the Sapporo General Pathology Laboratory (Sapporo, Japan).

Immunohistochemical staining for α-smooth muscle actin (α-SMA; D4K9N, Cell Signaling Technology, Danvers, MA, USA), and histological staining with Masson’s trichrome and von Kossa, were performed on formalin-fixed, paraffin-embedded heart tissue sections to evaluate smooth muscle content, fibrosis, and calcification, respectively. α-SMA staining was visualized with DAB and counterstained with hematoxylin, while von Kossa-stained slides were counterstained with nuclear fast red. Following staining, images used for quantification were acquired using a BZ-X800 microscope (Keyence, Osaka, Japan), and the extent of positive staining in the region surrounding the Valsalva sinus was measured using ImageJ software and expressed as a percentage of the arterial area.

The aortae were prepared for en face analysis to measure the lesion areas using an SMZ745T stereomicroscope (Nikon). Paraformaldehyde-suspended aortic arches (4%) were suspended in PBS for more than 24 h and then cut open in the coronal plane by introducing Vannas scissors into the aortic lumen to expose the intimal surface. The outer curvature of the ascending arch and its branches, including the brachiocephalic artery, were also dissected. Plaques in the aortic arch were stained using Sudan IV. The prepared samples were photographed using a COOLPIX S8100 camera (Nikon). Plaque areas were determined using the ImageJ software.

Oil Red O staining was performed on frozen liver sections to detect lipid droplets, followed by hematoxylin counterstaining. Quantitative assessment of lipid accumulation was conducted using stained images captured with BZ-X800 microscope. For each sample, five representative fields were analyzed using the ImageJ software, and the lipid-positive area was expressed as a percentage of the total area of the tissue.

Immunohistochemical staining of the liver tissue for B lymphocytes (CD45; RA3-6B2, BD Pharmingen, NJ, USA) and T lymphocytes (CD3; SP7, Nichirei Biosciences, Tokyo, Japan) was performed using specific secondary antibodies and DAB chromogen in frozen liver sections. Frozen sections were prepared and stained at Tokushima Molecular Pathology Institute (Tokushima, Japan). The prepared slides were analyzed using a BX51 microscope (Olympus).

Fluorescent antibody staining was performed on frozen liver sections to confirm the subpopulation of infiltrated lymphocytes in the liver. Frozen liver sections were fixed with 4% paraformaldehyde and incubated with antibody diluent with background-reducing components (DAKO/Agilent, Santa Clara, CA, USA) for 1 h as a blocking step. The sections were then incubated with antibodies. B cells were stained using two combinations: CD11b-Alexa488/CD19-BV421/IgD-APC and CD138-Alexa488/CD19-BV421/IgD-APC. T cells were dual-stained with CD4-APC and CD8-Alexa488. CD138-Alexa488 was purchased from R&D systems (Minneapolis, MN, USA). Other antibodies were all purchased from Biolegend. The prepared slides were analyzed using a FV3000 confocal microscope (Olympus).

### Flow cytometry analysis

Blood was collected, and peritoneal cells were isolated from mice (*n* = 10/group), as mentioned above. Erythrocytes were removed using erythrocyte lysis buffer (BD PharmLyse™ Lysing Buffer; BD Bioscience). Peripheral blood cells were stained with CD11b-FITC (BioLegend, San Diego, CA, USA) and Ly6G-APC (BioLegend) to determine the number of neutrophils and monocytes. CD3-PE (BioLegend) and B220-FITC (BioLegend) were used to detect T and B cells (Supplementary Fig. 1). MD-1 expression on B cells (B220-PE^+^; BioLegend) was assessed using a biotinylated anti-mouse MD-1 monoclonal antibody (previously established, clone 7G1)^39^, followed by staining with streptavidin-APC (Supplementary Fig. 2) (BioLegend).

Peritoneal cells were analyzed for B cells (CD19⁺), B cell subsets (B1a cells: CD5-PE⁺ [BioLegend], CD23-PECy7⁻ [eBioscience, SanDiego, CA, USA]; B1b cells: CD5-PE⁻, CD23-PECy7⁻; and B2 cells: CD5-PE⁻, CD23-PECy7⁺, calculated as a percentage of CD19-APC⁺ [BioLegend] cells) (Supplementary Fig. 3). Mast cells were identified as CD117-PE⁺ (BioLegend) and FcεRI-FITC⁺ (BioLegend) (Supplementary Fig. 4).

FACS analysis was performed using Novocyte (for mast cell analysis; Agilent, Santa Clara, CA, USA) or Fortessa (BD Biosciences), and the data were analyzed using NovoExpress (Agilent, for mast cells) or FlowJo software (Tree Star, Ashland, OR, USA).

### Intracellular cytokine staining

Splenocytes from LDLr^−/−^/MD-1^+/−^ and LDLr^−/−^/MD-1^−/−^ mice under normal chow diet (2 females and 2 males of siblings, between 18 and 25 weeks after birth) were stimulated with 20 ng/ml PMA and 2 µg/ml ionomycin in the presence of 20 μg/ml brefeldin A for 4 h at 37 °C. For cell surface staining, the cells were incubated with APC-conjugated anti-CD4 mAb (BioLegend) for 15 min at 4 °C. The cells were fixed and permeabilized using the True-Nuclear Transcription Factor Buffer Set (BioLegend) according to the manufacturer’s specifications, and then incubated with PE-conjugated anti-IL-4 and IFN-γ mAbs (BioLegend).

### Serum immunoglobulin detection

Serum was collected 24 weeks after HFD administration. Serum levels of IgG, IgG1, IgG3, IgG2b, IgG2c, IgA, IgM, and IgE were measured using ELISA. To detect Ig in the serum, 96-well plates were coated with goat anti-mouse IgG (Southern Biotech). Blocking was performed using a casein buffer. The plates were then incubated with serially diluted serum samples. After 1 h, the plates were incubated with HRP-conjugated goat anti-mouse IgG, IgG1, IgG3, IgG2b, IgG2c, IgA, IgM, and IgE (Southern Biotech). Finally, immunoglobulin levels were measured using TMB solution (Thermo Fisher Scientific, Waltham, MA, USA). Absorbance at 450 nm was measured using a Spectramax M5 microplate reader (Molecular Devices, San Jose, CA, USA).

### Statistical analysis

Data are expressed as mean ± SEM or as median. Datasets were analyzed using the Excel normality test in Statcel-the Useful Addin Forms on Excel-2nd ed. software. First, we determined whether the data were normally distributed. If one of the datasets was not normally distributed, non-parametric tests (Mann–Whitney U test) were used to calculate the *P*-values. For normally distributed data, either the Student’s *t*-test or Welch’s *t*-test was used to compare the groups, depending on whether the variances between the two groups were equal. Spearman’s correlation coefficient by rank test was used for the analysis of the correlation between two variables. *P*-values < 0.05 were considered statistically significant (n.s., not significant; *P < 0.05; ** P < 0.01; *** P < 0.001).

## Supplementary Information

Below is the link to the electronic supplementary material.

Supplementary Material 1

## Data Availability

The data are provided within the manuscript or in the supplementary information files.

## Abbreviations

HFD: High fat diet
MD-1: Myeloid differentiation-1
RP105: Radioprotective 105
