# Supplementary material for: Enhanced lymphocyte infiltration in the liver of LDL receptor and Myeloid Differentiation 1 double knockout mice on high fat diet
Source: Sci Rep. 2025 Oct 23;15:37077. doi: 10.1038/s41598-025-21070-x (PMC12549959; doi:10.1038/s41598-025-21070-x)
Supplement: Supplementary file 1 — Supplementary Material 1 [file 41598_2025_21070_MOESM1_ESM.pdf]

Enhanced lymphocyte infiltration in the liver of LDL receptor and Myeloid Differentiation 1 double knockout mice on high fat diet

Mrityunjoy Biswas[1], Susumu Tomono[1], Kenji Kasai[2], Hidekazu Takagi[1], Masanori Inui[1], Bristy Basak[1], Fumiaki Nagaoka[1], Tatsuya Yamazaki[1], Naoko Morita[1], Akinori Okumura[1], and Sachiko Akashi-Takamura[1*]

Supplementary Information

Fig.S1. Representative FACS results of monocytes, neutrophils, and B cells in peripheral cells, related to Figure 3a.
Fig.S2. Representative FACS results of MD-1 in peripheral cells, related to Figure 3a.
Fig.S3. Representative FACS results of B cells, B1a cells, and B1b cells in peritoneal cells, related to Figure 3b.
Fig.S4. Representative FACS results of mast cells in peritoneal cells, related to Figure 3b.
Fig.S5. Representative HE-stained liver sections of LDLr$^{-/-}$/MD-1$^{-/-}$ mice or LDLr$^{-/-}$/MD-1$^{+/-}$ mice and the percentage of lymphocyte infiltration area versus total liver area under (a) High Fat Diet for 24 weeks after 7 weeks of age and (b) Normal Chow Diet for 36-37 weeks.

Supplementary Table: The details of NAS score, related to Figure 5b.

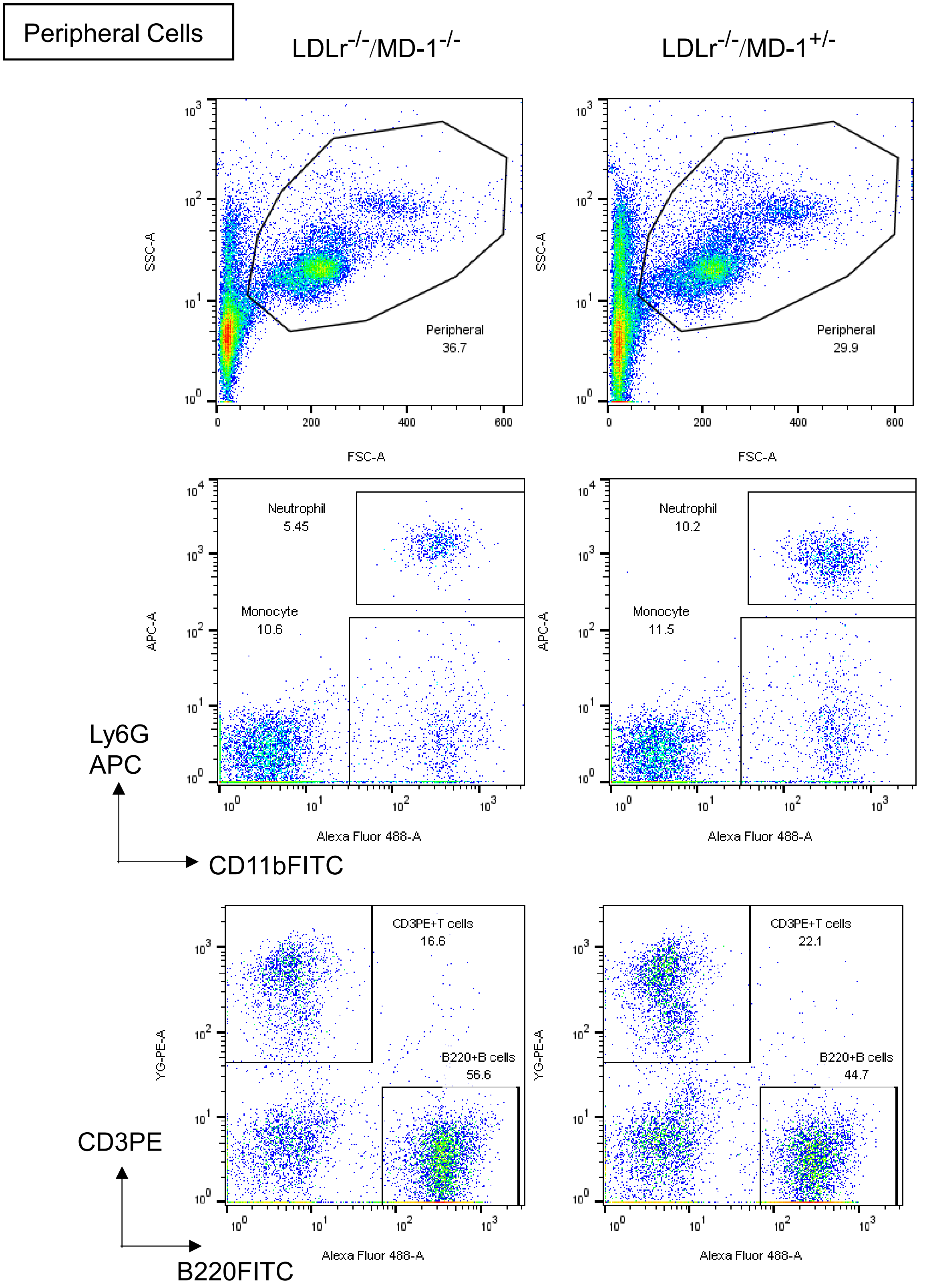

Supplementary Figure 1. Representative FACS results of monocytes, neutrophils, and B cells in peripheral cells, related to Figure 3a. Peripheral blood cells were stained with CD11b-FITC; Ly6G-APC to determine the amounts of neutrophils and monocytes. CD3-PE; B220-FITC; were used to detect T and B cells, respectively.

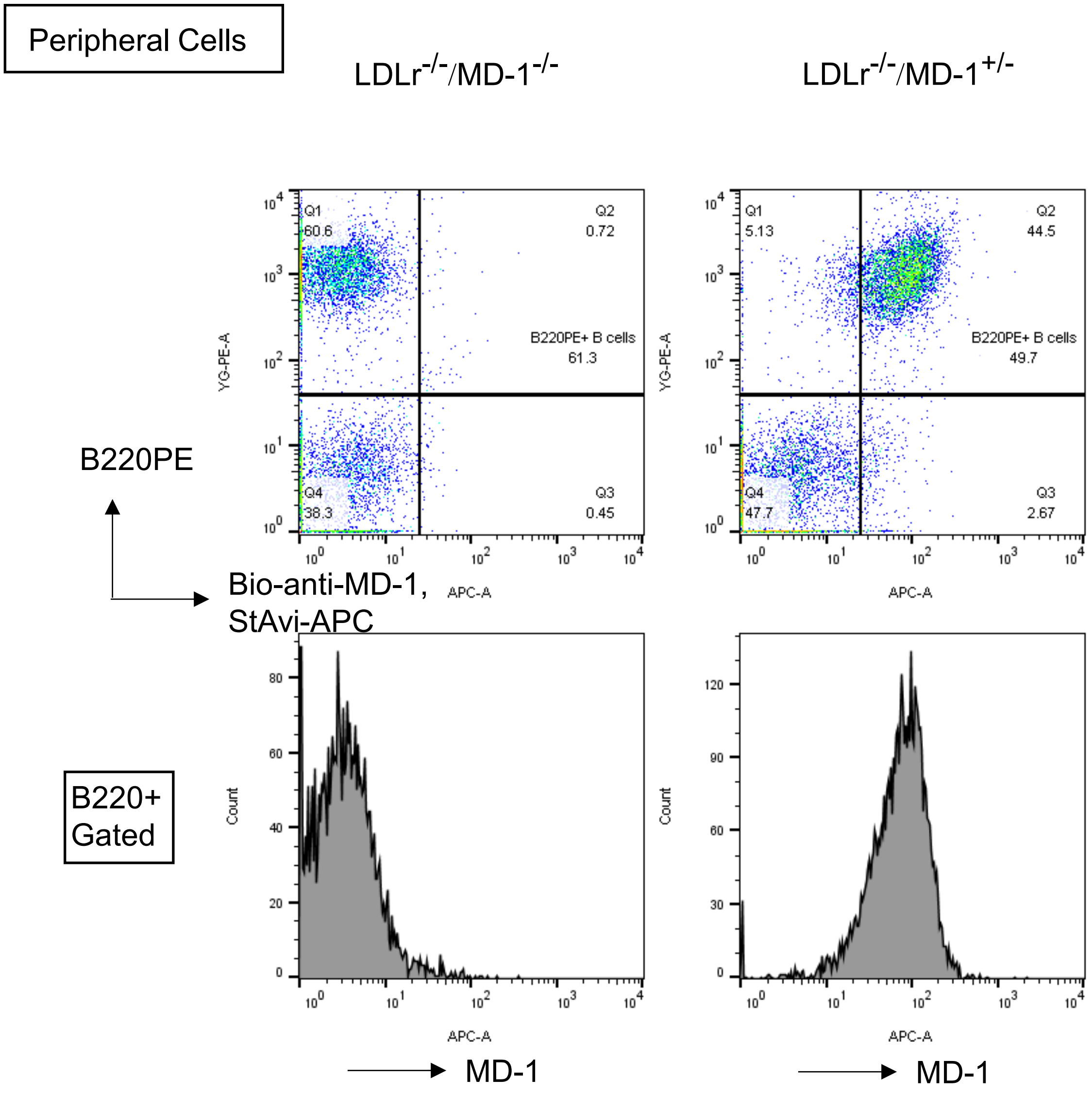

Supplementary Figure 2. Representative FACS results of MD-1 in peripheral cells, related to Figure 3a. MD-1 expression on B cells ($B220^{+}$) were analyzed by using biotinylated anti-mouse MD-1 mAb (previously we established (clone 7G1) (39)), followed by stained with streptavidin-APC (BioLegend, CA, USA) in peripheral blood cells.

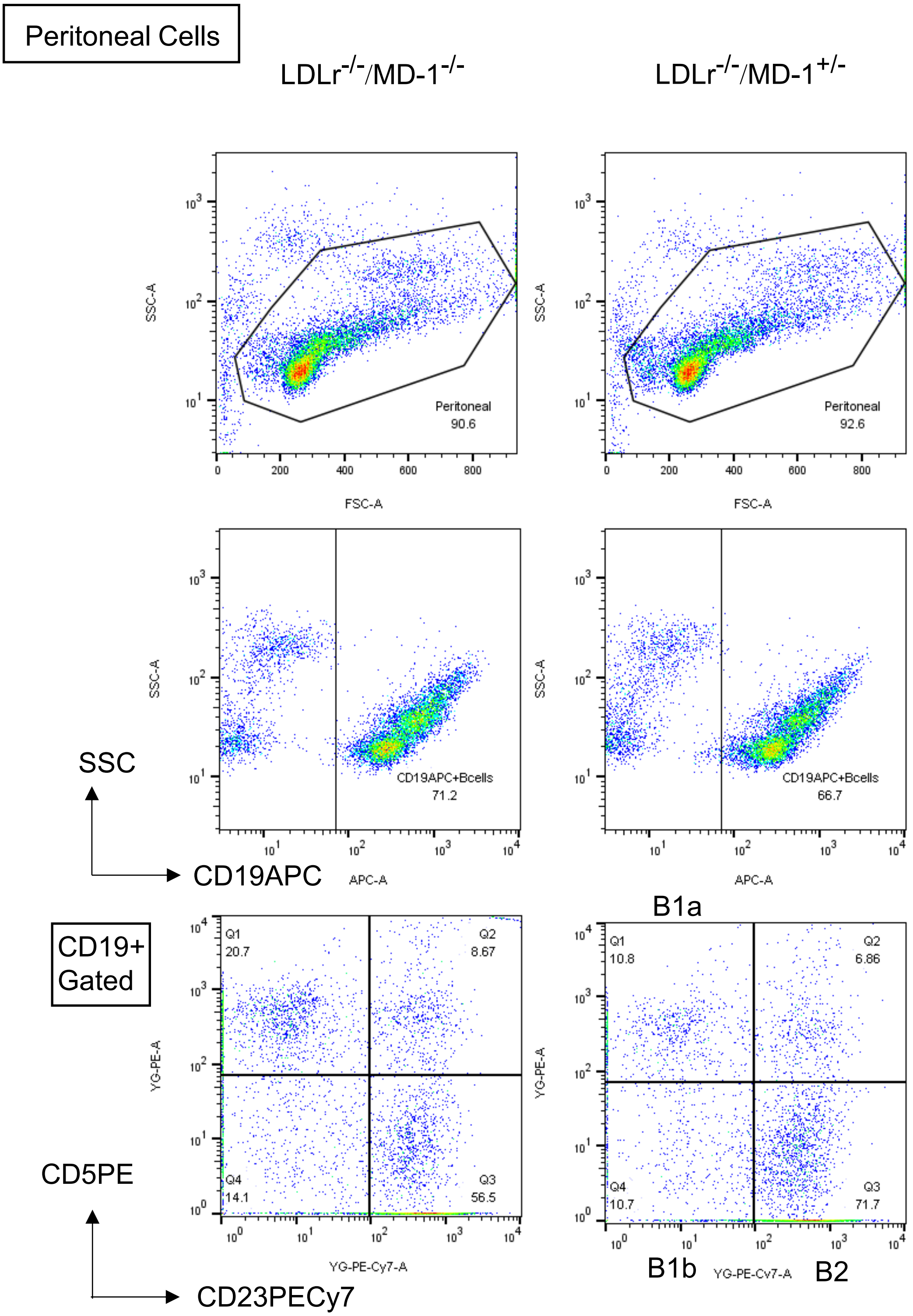

Supplementary Figure 3. Representative FACS results of B cells, B1a cells, and B1b cells in peritoneal cells, related to Figure 3b. Representative flow cytometry plots of peritoneal cells. Peritoneal cells were stained to analyze B cells ($CD19^+$), B cell subsets (B1a cells: $CD5^+$; $CD23^-$; B1b cells: $CD5^-$; $CD23^-$; and B2 cells: $CD5^-$; $CD23^+$; all calculated as a percentage of $CD19^+$ cells.

Peritoneal Mast Cells

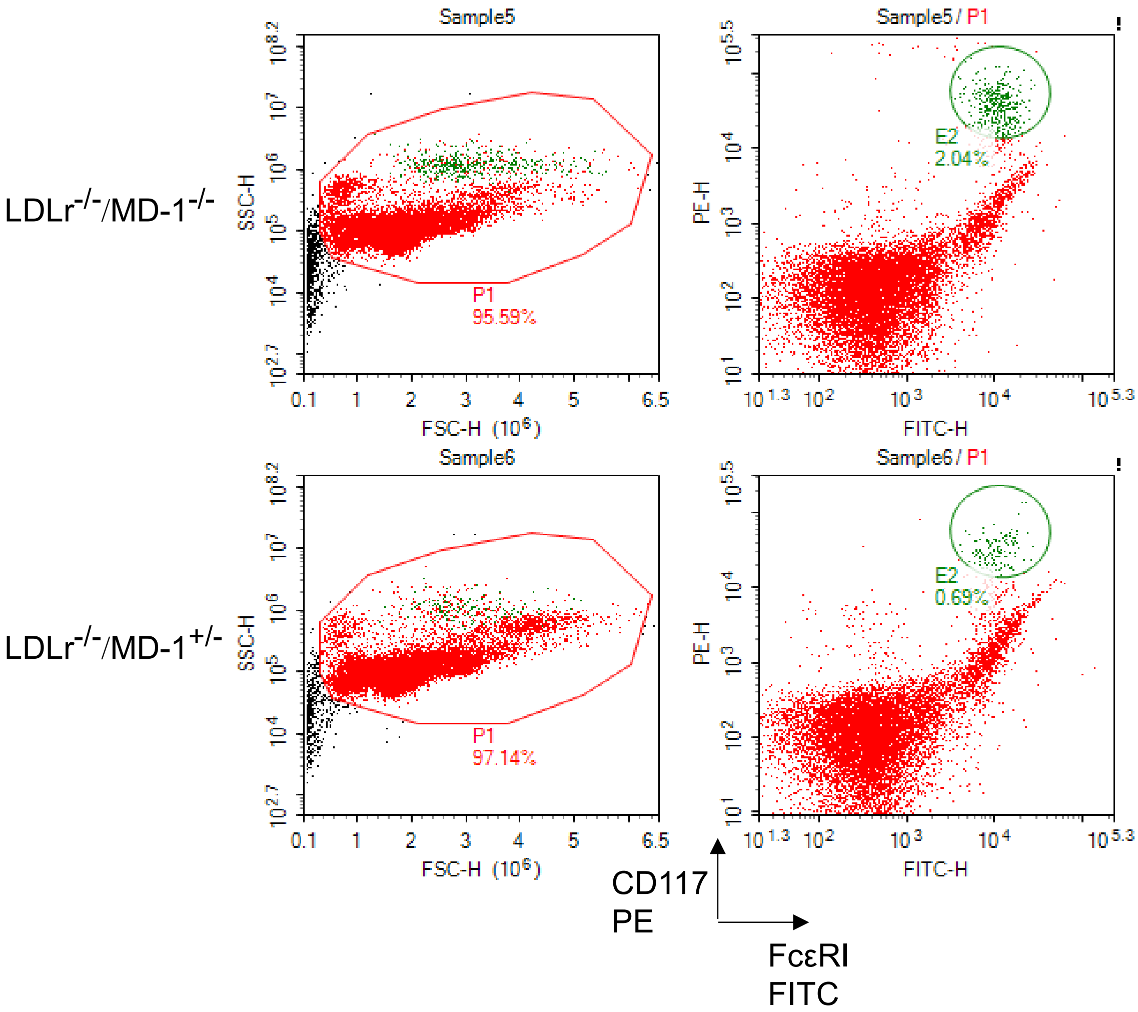

Supplementary Figure 4. Representative FACS results of mast cells in peritoneal cells, related to Figure 3b. Peritoneal cells were stained to analyze mast cell populations (E2 = $CD117^{+}$; $Fc\varepsilon RI^{+}$).

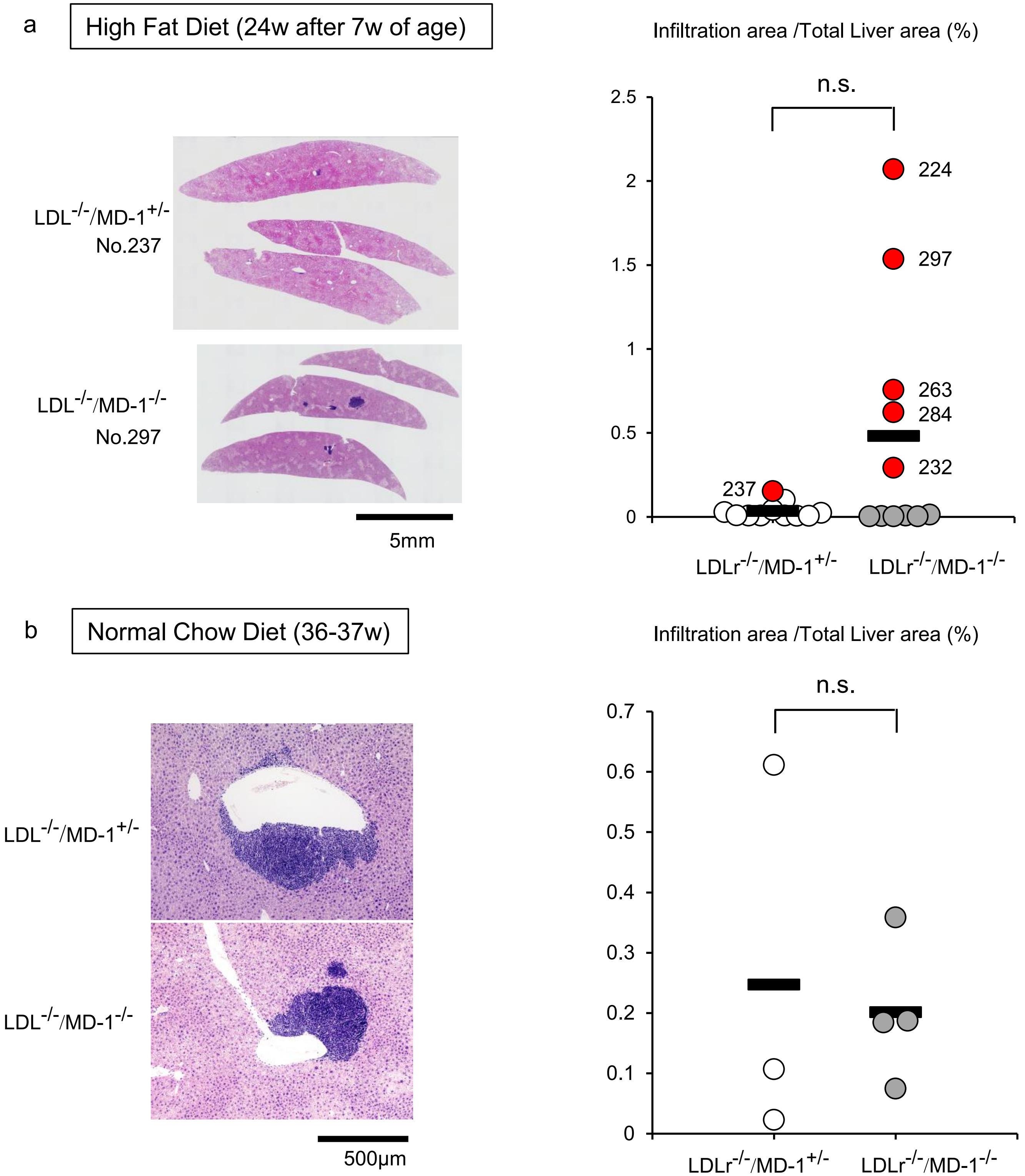

Supplementary Fig.5. Representative HE-stained liver sections of LDLr$^{-/-}$/MD-1$^{+/-}$ mice or LDLr$^{-/-}$/MD-1$^{-/-}$ mice and the percentage of lymphocyte infiltration area versus total liver area under (a) High Fat Diet for 24weeks after 7 weeks of age and (b) Normal Chow Diet for 36-37 weeks. (a) (Left): Image of the entire tissue section of Fig.6 mice No.237 and 297 (Scale bar = 5 mm). (Right): The percentage of lymphocyte infiltration area versus total liver area. Fig.6 samples are indicated as red mark with mice number. (n = 11). Data are presented as mean. n.s., not significant (Mann-Whitney's U test). (b) (Left): Image of the entire tissue section of Normal Chow Diet (Scale bar = 500 μm). (Right): The percentage of lymphocyte infiltration area versus total liver area. (LDLr$^{-/-}$/MD-1$^{+/-}$ mice: n = 3, LDLr$^{-/-}$/MD-1$^{-/-}$ mice: n = 4). Data are presented as mean. n.s., not significant (Student's t-test).

Supplementary Table. The details of NAS score, related to Figure 5b.

Score	Fatty deposites in hepatocytes	Balloon-like degeneration of hepatocytes	Inflammation within the hepatic lobules
0	< 5%	None	None
1	5% ~ 33%	Few	< 2 locations
2	33% ~ 66%	Many	2~4 locations
3	> 66%		> 4 locations
Code	Steatosis	Balloning	Inflammation
